# Association of umbilical cord blood lead with neonatal behavior at varying levels of exposure

**DOI:** 10.1186/1744-9081-2-22

**Published:** 2006-06-27

**Authors:** Archana B Patel, Manju R Mamtani, Tushar P Thakre, Hemant Kulkarni

**Affiliations:** 1 Clinical Epidemiology Unit, Indira Gandhi Government Medical College, Nagpur, India; 2 Lata Medical Research Foundation, Nagpur, India; 3 University of North Texas Health Science Center, Fort Worth, TX, USA

## Abstract

**Background:**

In the light of the ongoing debate about lowering the cut-off for acceptable blood lead level to <5 μg/dL from the currently recommended level of <10 μg/dL, we considered whether prenatal exposure to varying levels of lead is associated with similar or disparate effects on neonatal behavior.

**Methods:**

Using Brazelton's Neonatal Behavioral Assessment Scale (NBAS), an epidemiological approach and robust statistical techniques like multivariate linear regression, logistic regression, Poisson regression and structural equations modeling analyses we estimated the simultaneous indirect effects of umbilical cord blood lead (CBL) levels and other neonatal covariates on the NBAS clusters.

**Results:**

We observed that when analyzed in all study subjects, the CBL levels independently and strongly influenced autonomic stability and abnormal reflexes clusters. However, when the analysis was restricted to neonates with CBL <10 μg/dL, CBL levels strongly influenced the range of state, motor and autonomic stability clusters. Abnormal walking reflex was consistently associated with an increased CBL level irrespective of the cut-off for CBL, however, only at the lower cut-offs were the predominantly behavioral effects of CBL discernible.

**Conclusion:**

Our results further endorse the need to be cognizant of the detrimental effects of blood lead on neonates even at a low-dose prenatal exposure.

## Background

There is an ongoing debate over the appropriate cut-off of blood lead concentration to detect lead poisoning [1-6]. Starting from 60 μg/dL the cut-off recommended by the Centers for Disease Control (CDC) receded to 25 μg/dL and then to the currently used value of 10 μg/dL[5]. This was essentially due to a series of studies showing that even at low doses of exposure, environmental lead continues to be a biological and social toxicant [4,5,7,8]. Recently, there is a burgeoning recognition that even at low doses exposure to lead has serious implications on a child's behavior pattern. For example, lead exposure in low doses has been convincingly implicated in juvenile delinquency [9,10], intelligence quotient (IQ) patterns [4,11-18] and crime rates [19,20]. In the light of these findings, Needleman and others recommend that the time has arrived to lower the CDC recommended cut-off for blood lead to 5 μg/dL [5].

Blood lead has also been considered for a long time to be a behavioral teratogen. Interestingly, however, literature on the putative association of the prenatal blood lead exposure with the behavioral prototypes in the newborns is scant and inconsistent [2]. For example, Ernhart et al [21], Rothenberg et al [22] and more recently Emory et al [23] could not demonstrate any striking association between umbilical cord blood lead level and neonatal behavior. In contrast, two recent prospective studies have – using the Mental Development Index (MDI) – shown association of low-exposure to lead with the neurobehavioral development in early life [24,25]. Additionally, since neonatal behavior is a multi-dimensional construct with several hard-to-measure and correlated domains, the analytical strategy to test the association between blood lead levels and behavioral indicators is not always straightforward [2,26].

We therefore undertook this study to address two research questions: a) Do umbilical cord blood lead (CBL) levels independently correlate with the early neonatal neurobehavioral pattern? b) Do these neurobehavioral associations, if any, continue to be present in neonates with CBL levels below 10 μg/dL? We hypothesized that the behavioral archetypes of neonates are influenced by the level of prenatal exposure to lead even at relatively low doses of exposure. To test this hypothesis, we conducted a cross-sectional study assessing the association between umbilical cord blood lead levels and the neonatal neurobehavioral responses using appropriate measurement scales and statistical models.

## Methods

### Study subjects

The present cross-sectional study was conducted at the Government Medical College and Hospital, a tertiary care hospital in Nagpur, India. The data were collected over a four-month period starting from January 1998. All consecutively born neonates at the study center whose mother gave an informed consent were included in the study. Overall, 230 children were included. However, blood lead measurements were available on 176 (~77%) of the neonates who comprised our study sample. The study was approved by the Ethical Committee of the Government Medical College, Nagpur, India.

### Study variables

#### Outcomes

We measured the neonatal behavior using Brazelton's Neonatal Behavioral Assessment Scale (NBAS) [27]. The scale consists of the 28 behavior-related items scored on a 9-point scale, 18 reflexes and 7 supplementary items. Two trained pediatricians administered the scale. Before the study began, these two investigators independently and together evaluated a separate set of 20 neonates to ensure concordance of observations. The NBAS was administered within three days of birth. Since the arousal state can influence a newborn's performance on the individual items of the NBAS scale [27], we noted the initial state (the state of the newborn at the beginning of the NBAS evaluation) and predominant state (the state which the newborn was most commonly in over the duration of NBAS assessment and which was recorded at the end of the NBAS evaluation) of the newborn. We converted the raw scores on the NBAS items into the following seven clusters as recommended by Lester et al [28]: habituation, orientation, motor, range of state, regulation of state, autonomic stability and abnormal reflexes. The association of the predictor variables was then assessed with the cluster scores.

#### Blood lead measurement

Cord blood samples (5 ml) were obtained for each neonate in a metal-free K3 EDTA bulb and analyzed within 48 hours of sample collection for blood lead by flameless atomic absorption spectrophotometry (Hitachi Z-8000) in parts per billion at a wavelength of 283.3 nm with a slit width of 1.3 nm using the method described by Lagesson et al [29]. The detection rate of lead for the instrument was 1 μg/l, with an average error rate of 5% for reproducibility of results. The samples were analyzed for estimation of the lead concentration within 48 hours of collection.

#### Covariates

Table 1 describes the characteristics of the study subjects. In multiple linear regression analyses (described below), we used the following covariates: maturity, hours of birth, sex, birth weight, head circumference, fetal and maternal obstetric problems, specific disorder in fetus/newborn, problem noted during labor, use of oxytocic agents, rupture of membranes before onset of labor, tobacco intake by the mother and alcohol intake by the mother. The meaning and description of some of these covariates is provided in details in Supplementary Table 1 (see additional file 1, supplementary table 1). The covariates were measured based on the antenatal medical records, labor notes and by interviewing the mothers.

**Table 1:** Characteristics of the neonatal study subjects (n = 176)

Maturity in weeks (mean ± S.D)	38.93 ± 3.45
Hours of birth (mean ± S.D)	45.78 ± 15.15

Sex	
Male (n, %)	99 (56.3)
Female (n, %)	77 (43.7)

Birth weight (g, mean ± S.D)	2644.35 ± 413.15

Head circumference (cm, mean ± S.D)	32.47 ± 2.13

Umbilical cord blood lead level (μg/dL, mean ± S.D)	5.15 ± 12.65

Fetal obstetrical problem (n, %)	
Yes	6 (3.4)
No	157 (89.2)
Unknown	13 (7.4)

Specific disorder in fetus/neonate (n, %)	
Yes	17 (9.6)
No	145 (82.4)
Unknown	14 (8.0)

Maternal obstetrical problem (n, %)	
Yes	44 (25.0)
No	119 (67.6)
Unknown	13 (7.6)

Problem noted during labor (n, %)	
Yes	59 (33.5)
No	109 (61.9)
Unknown	8 (4.6)

Use of oxytoxic agents during labor (n, %)	
Yes	44 (25.0)
No	127 (72.2)
Unknown	5 (2.8)

Rupture of membranes before labor onset (n, %)	
No	135 (76.7)
Less than 24 hours	29 (16.5)
24 to less than 72 hours	5 (2.8)
72 to less than 120 hours	1 (0.6)
More than 120 hours	1 (0.6)
Unknown	5 (2.8)

Maternal medical problem during this pregnancy (n, %)	
Yes	30 (17.1)
No	137 (77.8)
Unknown	9 (5.1)

Tobacco intake by mother (n, %)	
No	164 (93.2)
Yes	8 (4.6)
Unknown	4 (2.2)

Alcohol intake by mother (n, %)	
Yes	3 (1.8)
No	169 (96.0)
Unknown	4 (2.2)

House painted (n, %)	
No or white wash	98 (55.7)
Yes, some	35 (19.9)
Yes, complete	39 (22.2)
Unknown	4 (2.2)

Age of house paint (n, %)	
< 5 years	62 (83.8)
5 – 10 years	7 (9.5)
Unknown	5 (6.7)

NBAS cluster scores (mean ± S.D)	
Habituation	28.91 ± 3.29
Orientation	43.06 ± 8.19
Motor	26.60 ± 3.69
Range of state	16.05 ± 3.83
Regulation of state	18.69 ± 5.38
Autonomic stability	14.12 ± 3.29
Abnormal reflexes	2.37 ± 1.98

### Statistical analysis

Our general strategy for statistical analysis was to test the association between cord blood lead levels and each NBAS cluster score in univariate and multivariate contexts. Since, in theory, the NBAS clusters represent essentially orthogonal i.e. uncorrelated factors, we used the score for each NBAS cluster as an outcome. For estimating the unadjusted influence, we used only CBL level as the predictor. Subsequently in a multiple linear regression model we estimated the adjusted influence of CBL for each NBAS cluster score by including the covariates mentioned above, the initial and predominant states of arousal (Table 2). It was essential to include both initial and predominant states in the multiple regression models because there two variables were not completely collinear with each other indicating that in a given infant often the initial state was not the same as the predominant state (Spearman's rho = 0.093, p = 0.1938). Lastly, only for the "abnormal reflexes" cluster we used single and multiple Poisson regression analyses because the scores for this cluster actually represent the count of the number of abnormal reflexes.

**Table 2:** Results of regression analyses for prediction of NBAS cluster scores based on CBL and other covariates^† ^in all neonates (left column) and neonates with CBL levels <10 μg/dL.

**NBAS cluster**	**All Neonates**		**Neonates with CBL < 10 μg/dL**	
	*Unadjusted *(coefficient, p)	*Adjusted *(coefficient, p)	*Unadjusted *(coefficient, p)	*Adjusted *(coefficient, p)

Habituation	0.0145, 0.468	0.0292, 0.213	-0.0432, 0.812	-0.0057, 0.988
Orientation	0.0092, 0.853	0.0176, 0.753	0.2823, 0.518	1.5972, 0.053
Motor	0.0188, 0.446	0.0108, 0.724	-0.2733, 0.136	0.4154, 0.282
Range of state	-0.0304, 0.196	-0.0419, 0.085	-0.5135, 0.008	-0.1957, 0.548
Regulation of state	0.0030, 0.930	0.0458, 0.336	-0.7138, 0.010	-1.2912, 0.036
Autonomic stability	-0.0567, 0.008	-0.0506, 0.077	-0.1219, 0.462	-0.3156, 0.507
Abnormal reflexes*	0.0118, 6.8 × 10^-5^	0.0073, 0.084	-0.0487, 0.163	-0.1049, 0.168

^† ^List of the covariates is provided in the Methods section, Study Variables subsection

* Estimated using Poisson regression analysis

Our next step of analysis was to assess the association of the CBL levels with the NBAS cluster scores in a multivariate context. For this purpose, we first conducted analysis of covariance (ANCOVA) using each NBAS cluster as the outcome and CBL as the predictor – first alone (unadjusted analysis) and then using initial and predominant states as covariates (adjusted analysis). Using the results from these analyses, we tested for the influence of blood lead on multiple outcomes using the *M*ultiple *I*ndicator *M*ult*i*ple *C*auses (MIMIC) model under the umbrella of Structural Equations Modeling (SEM). The details of the MIMIC model that we employed in our analyses are described below.

Additionally, we used Poisson regression to test the association between blood lead and the number of abnormal reflexes and multiple logistic regression analysis to test the association between various reflexes and dichotomized values of blood lead as described in the succeeding sections. We used Stata 8.0 (Stata Corp, College Station, TX) and Amos 5.0 (Amos Development Corp, Spring House, PA) for statistical analyses. Unless specified otherwise, an alpha error rate of 0.05 was used to test statistical significance.

## Results

The characteristics of the study subjects are described in Table 1 and Supplementary Table 1 (see additional file 1, supplementary table 1). Only two (1.1%) neonates were premature (<32 weeks), 10 (11.3%) had head circumference less than 30 cm, eight (4.6%) were small (birth weight < 2 kg), three (1.7%) were very small (birth weight < 1.5 kg) and 14 (8.0%) had cord blood lead exceeding 10 μg/dL. In general, therefore our study sample mostly included healthy neonates. This was also reflected by the mean scores for each of the NBAS clusters as shown in Table 1. During the NBAS evaluation, the most common initial states were light sleep (65 neonates, 36.9%), deep sleep (43 neonates, 24.4%) and alertness (35 neonates, 19.9%) while the most common predominant states were alertness (70 neonates, 39.7%), open eyes (49 neonates, 27.8%) and crying (31 neonates, 17.6%).

### CBL and NBAS cluster scores

The results shown in Table 2 indicated that when the analyses were conducted in all study subjects, the CBL levels significantly correlated with the autonomic stability and abnormal reflexes clusters even after adjustment for the aforementioned covariates. However, when the same analyses were performed in neonates with CBL levels <10 μg/dL, the unadjusted analyses identified the association of the CBL levels with the range of state and regulation of state clusters but the adjusted model identified the association with orientation and regulation of state clusters. We also considered whether the association of CBL with each NBAS cluster is specifically influenced by the potential effect of the initial and predominant states of the newborn on the NBAS cluster scores and found, using ANCOVA, that it was not (see additional file 1, supplementary table 2). This first pass analysis through the multiple regression models and ANCOVA thus indicated that i) The CBL levels correlated with specific NBAS clusters; ii) The CBL levels were differentially associated with NBAS clusters in all subjects versus subjects with CBL levels below 10 μg/dL and iii) The association of CBL levels with NBAS clusters varied between the unadjusted and adjusted analyses in neonates with low-dose prenatal lead exposure.

### Correlation among NBAS cluster scores

**Figure 1:**
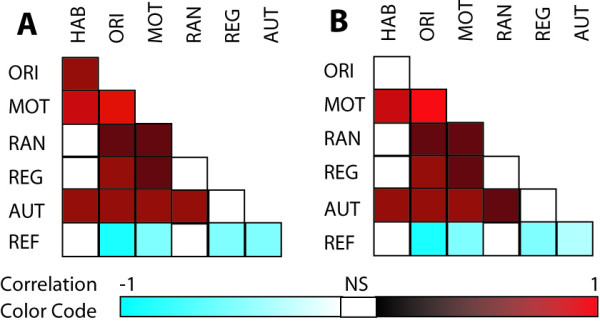
**Correlation structure of the NBAS cluster scores in all neonates (A) and neonates with the CBL levels below 10 μg/dL (B)**. The color codes at the bottom provide a reference for the magnitude and significance of the Pearson correlation coefficients. Open boxes represent statistically non-significant correlation coefficient. The actual estimates of correlation coefficients and their significance values are shown in Supplementary Table 3 (see additional file 1, supplementary table 3). The NBAS clusters shown here are: habituation (HAB), orientation (ORI), motor (MOT), range of state (RAN), regulation of state (REG) and autonomic stability (AUT) and abnormal reflexes (REF).

Even though the NBAS clusters are theoretically uncorrelated, we assessed if the correlations among these clusters were dataset-specific. To consider this possibility and the implications thereof, we first assessed the correlation structure of the seven NBAS clusters in all neonates as well as in neonates with CBL levels below 10 μg/dL (Figure 1 and additional file 1, supplementary table 3). Not surprisingly, we observed that there were a number of statistically significant correlations between pairs of NBAS clusters. Specifically, the habituation, orientation and motor clusters were strongly correlated with each other while the range of state and regulation of state clusters showed a trend towards a significant correlation with each other in all neonates as well as in neonates with CBL levels below 10 μg/dL. Arguably, this correlation structure can alter the interpretations regarding the simultaneous influence of the predictors on the NBAS clusters. Therefore, we chose to conduct further analyses in which we modeled the influence of CBL levels and other covariates simultaneously on the NBAS clusters.

### Simultaneous effects of CBL on neonatal behavior: specification of the MIMIC model

To be parsimonious, we wanted to select the most significant NBAS clusters that were least likely to be correlated with each other. For this purpose, using a reverse approach, we first used CBL levels as the outcome and the NBAS cluster scores as the predictors. We conducted stepwise linear regression with a strict retention probability criterion of 0.05. The clusters that were retained in the final model (see additional file 1, supplementary table 4) were motor, autonomic stability and abnormal reflexes in all neonates and range of state in neonates with CBL levels below 10 μg/dL. Therefore, we chose these four clusters as outcomes for modeling the simultaneous effects of CBL. This choice of the NBAS clusters was also consistent with the observed correlation structure since habituation and orientation were strongly correlated with the motor cluster while regulation of state was moderately correlated with the range of state.

We then chose four neonate-related predictors which we modeled as the covariates – CBL levels, head circumference, maturity and birth weight. There were three reasons for choosing this set of covariates. First, there exists literature support for a putative association of these covariates with NBAS cluster scores. Second, in a series of stepwise regression models in our dataset, these variables were consistently associated with one or more of the NBAS clusters (see additional file 1, supplementary table 5). Finally, as these variables can be considered to be of a continuous disposition, the correlation matrices to be used in structural equations modeling are more reliable and easier to construct and require no preprocessing of the data.

**Figure 2:**
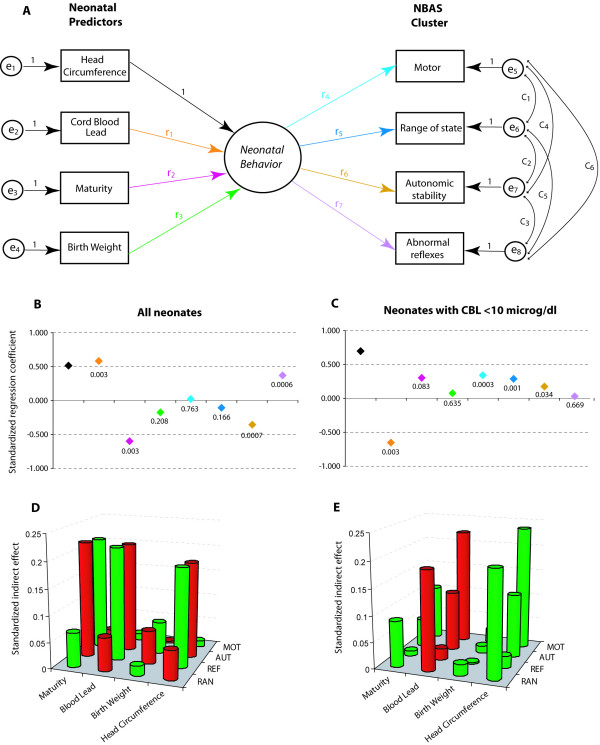
**Structural equations modeling of the influence of neonatal predictors on the NBAS clusters**. (**A**) The MIMIC model. The details of this model are given in text. Rectangles represent observed variables, circles represent latent variables, one-headed arrows represent influence and double-headed arrows represent covariance. The numbers or identifiers along the arrows are the model parameters. For ease of identification, the one-headed arrows of interest are color coded. Parameters e_1_–e_8 _represent the errors in measurement of observed variables. (**B and C**) Standardized regression coefficients for the color coded influences shown in panel A. Numbers indicate the statistical significance. The analysis was first conducted in all neonates (B) and then in neonates with CBL levels below 10 μg/dL (C). (**D and E**) Standardized indirect effects of the neonatal predictors (x-axis) on the NBAS cluster scores (y-axis). The z-axis represents the magnitude of the effect. Red cylinders indicate a negative effect while green cylinders indicate a positive effect. The analysis was conducted in all neonates (D) and then in neonates with CBL levels below 10 μg/dL (E). Complete results of SEM are shown in Supplementary Table 6 (see additional file 1, supplementary table 6). Abbreviations for the NBAS clusters are: motor (MOT), range of state (RAN), autonomic stability (AUT) and abnormal reflexes (REF).

The path diagram of our model (Figure 2A) thus contained four predictors and four outcomes. In SEM, a model of this nature is referred to as the *M*ultiple *I*ndicator *M*ult*i*ple *C*auses (MIMIC) model [28]. In the proposed MIMIC model, none of the predictors directly influences any of the outcomes, that is, there exists no direct arrow in the path diagram (Figure 2A) from any predictor to any outcome – they all pass through a conceptual, latent and unmeasured variable. We argue that these four predictors influence a latent (unobserved) trait which we refer to as the "neonatal behavior". In our model, the NBAS clusters were thus considered as indicators of the neonatal behavior.

We modeled the influence of the predictors on neonatal behavior and on the four outcomes within the framework of structural equations modeling (SEM). The regression weights (parameters labeled as r_1 _to r_7 _in Figure 2A) thus measure the influence of the predictors on each outcome in a multivariate context. The random errors of measurement associated with all observed variables – four predictors and four outcomes – were included as shown (parameters labeled as e_1 _to e_8 _in Figure 2A). Since the measurements of NBAS clusters are correlated, we assume that the measurement errors associated with these variables will also be correlated (shown by the curved arrows in the model and the parameters labeled as c_1 _to c_6_). Finally, to make the model identifiable, we constrained the head circumference → neonatal behavior regression weight to unity.

### Results from the MIMIC model

Figure 2B–E and Supplementary Table 6 (see additional file 1, supplementary table 6) show the results of SEM analyses using the MIMIC model. As the predictor and outcome variables are measured on different metrics, we present the data in the form of standardized estimates of the regression coefficients (Figure 2B and 2C). We observed that when the analysis was conducted in all neonates, CBL levels and maturity independently influenced neonatal behavior – more mature neonates had a better behavior score. Interestingly, this influence of CBL and maturity was detectable only with respect to autonomic stability and abnormal reflexes – the other two outcomes were not influenced. This analysis thus recaptured the observations from the previous analysis that even in a multivariate and multiple-outcome context the independent influence of CBL on autonomic stability and abnormal reflexes was discernible.

When the analysis was restricted to neonates with CBL levels below 10 μg/dL, we observed a notable shift in the pattern of association. The CBL levels were now the only statistically significant predictor and the influence on the neonatal behavior was limited to the motor, range of state and autonomic stability clusters. Thus, concordant with the earlier results, our results of MIMIC modeling reaffirmed that the dominant effects of CBL were different in all neonates compared to neonates with low-dose exposure to lead.

Our results of the SEM modeling indicated that the model fit was not adequate either for all neonates or for neonates with CBL <10 μg/dL. We further investigated the reason for this apparent lack of fit for which purpose we assessed the predictive performance of 10 other models nested within the model shown in Figure 2A. While constructing the nested models, we considered all combinations of the four predictors taken three at a time and then taken two at a time. These 10 models and their performance is shown (see additional file 1, supplementary table 7) in Supplementary Tables 7A (for all neonates) and 7B (for neonates with CBL <10 μg/dL). A close look at the model fits for these nested models revealed the following: i) Removal of CBL as a predictor from the MIMIC model always worsened the model fit; ii) Inclusion of maturity and head circumference was most of the times associated with a poor fitting model; iii) The best model for all neonates was with two predictors: CBL and maturity; and iv) The best model for neonates with CBL <10 μg/dL contained CBL and head circumference. Thus, the full model with all four predictors was associated with a poor model fit but we have shown it here only because it permitted us to study the effects of CBL adjusted for other potential confounders.

### Association of increased CBL levels on items within the significantly associated NBAS clusters

Given the significant multivariate effects of CBL levels on the four NBAS clusters included in the MIMIC model analyses in the previous step, we next considered whether there were any specific items within these clusters that were associated with the risk of increased CBL levels. For this purpose, we dichotomized the CBL levels into high and low using three different cut-off points: 5, 10 and 25 μg/dL. Using each of these binary outcomes we used backward stepwise unconditional multiple logistic regression analyses with a probability criterion of 0.05 to identify the NBAS items most significantly associated with the likelihood of possessing CBL levels exceeding these cut-offs. The results of these analyses are shown in Table 3.

**Table 3:** Association of NBAS items with risk of possessing high CBL levels: results from final models using stepwise multiple logistic regression analyses.

NBAS Item	Risk of CBL levels > the shown cut-off point		
	
	***OR***	***95% CI***	***P***
**5 μg/dL**			

*Range of State cluster*			
Peak of excitement	0.60	0.37 – 0.98	0.042
*Autonomic stability cluster*			
Tremors	0.77	0.63 – 0.94	0.012
*Abnormal reflexes*			
Moro's reflex	3.37	1.13 – 10.04	0.029
Walking reflex	3.55	1.24 – 10.15	0.018

**10 μg/dL**			

*Autonomic stability cluster*			
Tremors	0.75	0.58 – 0.96	0.023
*Abnormal reflexes*			
Babinski sign	4.26	1.01 – 17.8	0.047
Walking reflex	5.99	1.44 – 24.9	0.014

**25 μg/dL**			

*Abnormal reflexes*			
Babinski sign	11.3	1.89 – 68.1	0.008
Walking reflex	8.17	1.36 – 49.2	0.022

We observed that not all items within each cluster were significantly associated with the risk of an increased CBL level. For example, if a high value (>25 μg/dL) for the CBL cut-off was used then only two abnormal reflexes – Babinski's sign and walking reflex – were significantly associated. At the currently used cut-off of >10 μg/dL, tremors were additionally identified to be significantly associated, while at a lower cut-off of >5 μg/dL, the peak of excitement also was significantly associated. Moreover, Moro's reflex rather than Babinski's sign was the significant abnormal reflex.

## Discussion

In the process of human brain development the perinatal period characterizes a critical interval during which there is highest rate of brain development, rampant genesis of new synapses, widespread neuronal proliferation, and maximum density of the N-methyl-D-aspartate (NMDA) receptors [31-37]. The last of these facts bears a special relevance to lead neurotoxicity since it has been argued that the Ca^++ ^permeable NMDA receptors also act as the neuronal gateway for Pb^++ ^[38]. Therefore the newborn brain is especially prone to the toxic effects of environmental neurotoxicants [26] and can be expected to be sensitive to even low doses of lead exposure. Based on this biological rationale, using the NBAS administered within three days of birth and employing multivariate statistical approaches for analysis, we observed that umbilical cord blood lead levels were significantly associated with different aspects of the neonatal behavior even at relatively low doses of exposure.

### Study findings

We observed that the association of CBL levels with NBAS clusters was differential in two respects. First, not all NBAS clusters were equally associated with the CBL levels. Biologically, since the development of the newborn brain is neither simultaneous nor equivalent across all areas [26,39-41]; it can be expected that the influence of lead may not be alike on all areas of the developing brain. Indeed, several experimental studies have demonstrated that in the rat models of lead toxicity, the predominantly affected brain areas include the hippocampus [42,43], the hypothalamus [44], the prefrontal cortex [45], the temporal cortex [46] and the cerebellum [47]. In humans, the posterior hippocampus has been shown to be associated with behavior [48], the prefrontal cortex is known to control cognitive functions like language, abstract reasoning, problem solving, social interactions, and planning [49,50], the temporal lobe along with portions of hippocampus and prefrontal cortex has been implicated in object working memory [51] while cerebellum is the known seat of locomotion control. Our findings that the motor, range of state, autonomic instability and the abnormal reflexes NBAS clusters were specifically associated with the CBL: i) corroborate the conjecture that all domains of neonatal behavior will not be equally influenced by exposure to lead; and ii) are consistent with the known behavior-related functions of those areas in the human brain that have been shown in animal studies to be the primary targets for the effects of exposure to lead.

Second, and more interestingly, we found that the NBAS clusters associated with CBL levels in all neonates were not the same as the NBAS clusters identified by restricting the analyses to low levels of exposure. In neonates with CBL <10 μg/dL, we did not observe an association of the varying CBL levels with the abnormal reflexes cluster but did uncover an association with the motor cluster. These data indicate that relatively higher values of CBL will be needed for lead to demonstrate its influence on the abnormal reflexes; however at a relatively milder dose it may continue to demonstrate an association with the motor, autonomic instability and range of state clusters. Evidence to support the deleterious effects of low-dose lead exposure on human neonatal behavior is continuously increasing [24,25,52,53] however a novel finding of the present study is that the patterns of behavior are different in neonates with CBL <10 μg/dL as compared to those with a higher dose of exposure.

### Study limitations

Our study suffers from three limitations. First, for the reasons explained earlier, the main focus of our study was the behavioral patterns in the newborn which we assessed using NBAS. However, this is a cross-sectional study design – a fact that does not permit inferences about the potential causal role of low-dose lead exposure [25,54,55].

Second, a single measurement of umbilical cord blood lead is unlikely to faithfully capture the overall cumulative exposure to lead [25] thereby making our measurement of lead exposure questionable. We did not have data on serial measurements of the lead concentrations in mother's blood over the entire duration of pregnancy. Our rationale for using umbilical CBL was based on the following observations: i) As reported by previous studies, the correlation coefficient between maternal and umbilical cord blood lead levels ranges between 0.55 to 0.92 [56,57]; ii) All through gestation, lead is known to cross the placenta and is considered to be the most important source of umbilical cord blood lead [58]; and iii) independent of the maternal bone lead – an index of the cumulative lead exposure – umbilical cord blood lead has been shown to be a significant predictor of child development [25]. Considering these pieces of evidence from the literature and the absence of serial measurements of maternal blood lead in our study, we used umbilical cord blood lead as a surrogate for the cumulative lead exposure of the newborn.

Third, we did not have data on co-exposure of the newborn to other toxicants like cadmium and polychlorinated biphenyls which can also imitate some of the effects of lead [54,59,60]. In the absence of this data, our study will not be able to definitively point towards a causal role of lead, however the compatibility of our findings with the existing literature and the robust analytical methods used in this study urge the consideration of a plausible role of low-dose lead exposure in determining the patterns of neonatal behavior.

### Study implications

With the caveats mentioned in the preceding section, we believe that our study has three important implications. First, it is not currently known whether the neonates who are affected by the low levels of lead exposure grow into children more likely to be affected with regards to their overall mental health. However, it has been observed that children exposed to low doses of lead show suboptimal cognitive functioning and reduced intelligent quotients [12-14]. Further, the following observations indirectly suggest a strong link between the events in early neonatal life and childhood development: gestational low-dose exposure to lead in rats can lead to a significant future risk of alterations in monoaminergic metabolism during adulthood [61]; neonatal infection can result in robust hippocampal-dependent memory impairment in adulthood [62]; neonatal prefrontal cortex lesions can manifest in adult animals as behavioral disturbances [63]; and early life does have an influence on the behavioral patterns in later life [64]. Considering all these observations together, it is conceivable that neonates demonstrating behavioral disturbances secondary to lead exposure may continue to manifest these disturbances in childhood.

We believe that, among others, a possible reason for the discordance in the results and interpretations of the effects of low-dose lead exposure on neonatal behavior can be attributed to a lack of a standardized analytical protocol. Theoretically, lead can have multiple and simultaneous effects and we suggest that future studies need to incorporate statistical techniques like SEM to handle the data more efficiently and accurately. The use of Generalized Estimating Equations (GEE) for regressing the predictors [65] on multiple outcomes is another attractive alternative. In either case, the emphasis needs to be laid on the measurement and identification of a concomitant influence of blood lead – alone or with other predictors – on multiple outcomes related to behavior.

Another area of interest in the field of lead poisoning relates to the policies and practice of screening. Sargent and others [66] argue that in order to reduce the false positive error rate, it may be unwarranted to screen for children with blood lead levels between 10 and 15 μg/dL. As an alternative, Binns et al [67] suggested high-risk population screening. In situations where blood lead tests may not be easily or inexpensively available, it has also been thought to consider the use of blood lead questionnaires [68,69]. In that vein, we identified only a few NBAS items to be specifically correlated with the risk of possessing high CBL levels. Our findings imply that peak of excitement, tremors and abnormal Babinski's sign and walking reflexes may together serve as a potential initial screen to identify neonates possessing moderate to high CBL levels. While our study was not designed to address the issue of screening for lead toxicity, our results suggest that neonates with the aforementioned characteristics may need a further evaluation with a special emphasis on lead poisoning.

## Conclusion

Needleman [4] believes that we are now into a "fifth cycle" of understanding the effects of this commonest environmental toxicant. Our findings concur with the observation that the effects of reduced levels of blood lead only indicate a possible avoidance of the physical presentation of lead poisoning; they may not however preclude the more subtle behavioral repercussions that can continue to have a high impact on the social realm of the disease. Therefore efforts to reduce exposure to this physiologically redundant but environmentally toxic metal need to continue.

## Abbreviations

CBL Cord Blood Lead

NBAS Neonatal Behavioral Assessment Scale

SEM Structural Equations Modeling

MIMIC Multiple Indicators, Multiple Causes

ANCOVA Analysis of covariance

CDC Centers for Disease Control

IQ Intelligence quotient

## Competing interests

The author(s) declare that they have no competing interests.

## Authors' contributions

ABP conceptualized the study, participated in data collection, data management and the review of the manuscript. MRM contributed to the statistical analyses, created the illustrations, and contributed to the manuscript writing and review. TPT contributed to the review of the manuscript. HRK conducted the statistical analyses and wrote the manuscript. All authors read and approved the final version of the manuscript.

## Supplementary Material

Additional file 1 This MS Word file contains the seven Supplementary Tables alluded to in this manuscript. Supplementary Table 1 describes some covariates listed in Table 1 of the main text in more details. Supplementary Table 2 shows the results of ANCOVA analyses. Supplementary Table 3 shows the correlation coefficients between pairs of NBAS clusters and their statistical significance. The analysis is first shown in all neonates and then in neonates with CBL <10 mg/dL. Supplementary Table 4 shows the results of final models from stepwise multiple linear regression models predicting the CBL levels based on the NBAS cluster scores. Supplementary Table 5 shows the results of the final models from stepwise multiple linear regression analyses of the covariates on each of the four NBAS clusters chosen for SEM analysis. Supplementary Table 6 shows the results of the full SEM model. Lastly, Supplementary Table 7 shows the model fit indices for a total of 11 nested models. In Supplementary Tables 3 and 5 – 7, the analyses are first shown for all neonates and then for neonates with CBL <10 μg/dL. Click here for file
