# Supplementary material for: Association of umbilical cord blood lead with neonatal behavior at varying levels of exposure
Source: Behav Brain Funct. 2006 Jun 27;2:22. doi: 10.1186/1744-9081-2-22 (PMC1557521; doi:10.1186/1744-9081-2-22)
Supplement: Additional file 1 — This MS Word file contains the seven Supplementary Tables alluded to in this manuscript. Supplementary Table 1 describes some covariates listed in Table 1 of the main text in more details. Supplementary Table 2 shows the results of ANCOVA analyses. Supplementary Table 3 shows the correlation coefficients between pairs of NBAS clusters and their statistical significance. The analysis is first shown in all neonates and then in neonates with CBL <10 mg/dL. Supplementary Table 4 shows the results of final models from stepwise multiple linear regression models predicting the CBL levels based on the NBAS cluster scores. Supplementary Table 5 shows the results of the final models from stepwise multiple linear regression analyses of the covariates on each of the four NBAS clusters chosen for SEM analysis. Supplementary Table 6 shows the results of the full SEM model. Lastly, Supplementary Table 7 shows the model fit indices for a total of 11 nested models. In Supplementary Tables 3 and 5 – 7, the analyses are first shown for all neonates and then for neonates with CBL <10 μg/dL. [file 1744-9081-2-22-S1.doc]

**Supplementary Table 1.** Additional description of some covariates shown in table 1 (main text) and some additional characteristics.

|  | N |
| --- | --- |
| *Fetal obstetric problem (N=6)* |  |
| Oligohydramnios | 1 |
| Persistent abnormal presentation | 2 |
| Unspecified | 3 |
| *Specific disorder in fetus/newborn (N=17)* |  |
| Severe intrauterine growth retardation | 4 |
| Anemia | 3 |
| Congenital malformation | 2 |
| Excessive drug administration | 2 |
| Distress due to placenta previa | 1 |
| Infection | 1 |
| Distress during labor | 1 |
| Unspecified | 5 |
| *Maternal obstetric problem (N=44)* |  |
| Previous caesarean section | 28 |
| *History of problem in previous pregnancy | 11 |
| Placenta previa | 4 |
| Accidental hemorrhage | 1 |
| *Problem noted during labor (N=59)* |  |
| Cephalo-pelvic disproportion | 25 |
| Maternal/fetal distress | 14 |
| Obstructed labor | 9 |
| Cervical dystocia | 2 |
| Prolonged labor | 1 |
| Ruptured uterus | 1 |
| Unspecified | 7 |
| *Maternal medical problem during this pregnancy (N=30)* |  |
| Hypertensive disease of pregnancy | 18 |
| Anemia | 6 |
| Thalassemia / sickle cell anemia | 1 |
| Eclampsia | 1 |
| Hepatitis B | 1 |
| Malaria | 1 |
| Heart disease | 1 |
| Unspecified | 1 |
| *Caste (N=176)* |  |
| Mahar | 43 |
| Maratha, Kunbhi, Kshatriya | 38 |
| Teli | 20 |
| Tribal | 12 |
| Brahmin | 4 |
| Others | 53 |
| Unknown | 6 |
| *Mother’s education (N=176)* |  |
| High school | 90 |
| Middle school | 35 |
| Primary school | 16 |
| College graduate | 16 |
| Illiterate | 7 |
| Post graduate | 5 |
| Diploma | 3 |
| Unknown | 4 |

* Presence in previous pregnancies of the following: abortions or still birth, any one of the above stated fetal

obstetric problems, maternal obstetric problems or problems in labor.

**Supplementary Table 2**. Results of the Analysis of Covariance (ANCOVA) for prediction of each NBAS cluster by CBL alone (unadjusted) and CBL with initial and predominant states (adjusted). Left column is for all neonates included in the study while the right column is restricted to neonates whose CBL levels were below <10μg/dL. The numbers in cell are the F statistic and its significance value. The concordance between unadjusted and adjusted values shows that the effects of CBL were minimally influenced by the effects of initial and predominant states.

| NBAS Cluster | All neonates | | Neonates with CBL<10μg/dL | |
| --- | --- | --- | --- | --- |
|  | Unadjusted | Adjusted | Unadjusted | Adjusted |
| Habituation | 0.53, 0.4682 | 0.66, 0.4167 | 0.06, 0.8115 | 0.18, 0.6712 |
| Orientation | 0.03, 0.8532 | 0.05, 0.8290 | 0.42, 0.5178 | 0.38, 0.5379 |
| Motor | 0.58, 0.4460 | 0.34, 0.5619 | 2.25, 0.1361 | 0.03, 0.8743 |
| Range of state | 1.69, 0.1956 | 1.46, 0.2291 | 7.19, 0.0082 | 2.91, 0.0905 |
| Regulation of state | 0.01, 0.9299 | 0.00, 0.9840 | 6.92, 0.0095 | 9.49, 0.0026 |
| Autonomic stability | 7.13, 0.0084 | 3.65, 0.0581 | 0.54, 0.4620 | 0.04, 0.8425 |
| Abnormal reflexes | 10.6, 0.0014 | 14.5, 0.0002 | 1.19, 0.2775 | 0.24, 0.6215 |

**Supplementary Table 3.** Matrix of Pearson’s correlation coefficients between pairs of the NBAS cluster scores. Cells show correlation coefficients (regular font) and the respective significance value (small italic font).

***(A) All neonates***

| NBAS Cluster | HAB | ORI | MOT | RAN | REG | ANS |
| --- | --- | --- | --- | --- | --- | --- |
| ORI | 0.2048 |  |  |  |  |  |
|  | *0.0275* |  |  |  |  |  |
| MOT | 0.3641 | 0.5890 |  |  |  |  |
|  | *<0.0001* | *<0.0001* |  |  |  |  |
| RAN | 0.0998 | 0.1856 | 0.1667 |  |  |  |
|  | *0.2822* | *0.0275* | *0.0473* |  |  |  |
| REG | 0.1536 | 0.2240 | 0.1947 | 0.1590 |  |  |
|  | *0.1042* | *0.0095* | *0.0253* | *0.0655* |  |  |
| ANS | 0.2352 | 0.2918 | 0.2316 | 0.2177 | 0.1200 |  |
|  | *0.0118* | *0.0006* | *0.0067* | *0.0112* | *0.1757* |  |
| ABR | 0.0801 | -0.3258 | -0.2848 | -0.0317 | -0.2472 | -0.2206 |
|  | *0.3885* | *0.0001* | *0.0006* | *0.7069* | *0.0039* | *0.0091* |

***(B) Neonates with CBL<10μg/dL***

| NBAS Cluster | Habituation | Orientation | Motor | Range of state | Regulation of state | Autonomic stability |
| --- | --- | --- | --- | --- | --- | --- |
| Orientation | 0.1919 |  |  |  |  |  |
|  | *0.0522* |  |  |  |  |  |
| Motor | 0.3727 | 0.6190 |  |  |  |  |
|  | *0.0001* | *<0.0001* |  |  |  |  |
| Range of state | 0.1163 | 0.1793 | 0.1807 |  |  |  |
|  | *0.2330* | *0.0413* | *0.0381* |  |  |  |
| Regulation of state | 0.1703 | 0.2329 | 0.1884 | 0.1739 |  |  |
|  | *0.0871* | *0.0098* | *0.0377* | *0.0524* |  |  |
| Autonomic stability | 0.2851 | 0.3152 | 0.3138 | 0.1940 | 0.1297 |  |
|  | *0.0035* | *0.0003* | *0.0003* | *0.0295* | *0.1579* |  |
| Abnormal reflexes | 0.0234 | -0.3702 | -0.3167 | 0.0065 | -0.2747 | -0.1871 |
|  | *0.8104* | *<0.0001* | *0.0002* | *0.9405* | *0.0019* | *0.0324* |

**Supplementary Table 4.** Results of final models from stepwise multiple linear regression models predicting the CBL levels based on the NBAS cluster scores. The four NBAS clusters retained in the two models shown below were included in the MIMIC model for structural equations modeling.

| NBAS Cluster | Coefficient | SE | T | P |
| --- | --- | --- | --- | --- |
| ***(A) All neonates*** | | | | |
| Motor | 0.8803 | 0.3753 | 2.35 | 0.022 |
| Autonomic stability | -0.9816 | 0.4093 | -2.40 | 0.019 |
| Abnormal reflexes | 1.7806 | 0.7843 | 2.27 | 0.026 |
| *Intercept* | -9.3084 | 11.4518 | -0.81 | 0.419 |
| ***(B) Neonates with CBL<10μg/dL*** | | | | |
| Range of state | -0.1135 | 0.0524 | -2.17 | 0.034 |
| *Intercept* | 3.5006 | 0.9008 | 3.89 | 0.0003 |

**Supplementary Table 5**. Results of the final models from stepwise multiple linear regression analyses of the covariates on each of the four NBAS clusters chosen for SEM analysis in (A) All neonates and (B) Neonates with CBL<10μg/dL. The models used a probability retention criterion of 0.1.

**(A) All Neonates**

| Predictor | Coefficient | SE | T | P |
| --- | --- | --- | --- | --- |
| ***Motor*** | | | | |
| Maternal medical problem | -2.30 | 0.93 | -2.49 | 0.015 |
| Birth weight | 0.0038 | 0.0009 | 4.24 | <0.001 |
| Maternal obstetric problem | -1.57 | 0.83 | -1.90 | 0.062 |
| ***Range of state*** | | | | |
| Maternal obstetric problem | 1.61 | 0.76 | 2.13 | 0.037 |
| Duration of house paint | -1.54 | 0.49 | -3.14 | 0.002 |
| Birth weight | -0.0018 | 0.0008 | -2.17 | 0.034 |
| ***Autonomic stability*** | | | | |
| Maternal obstetric problem | -1.57 | 0.86 | -1.83 | 0.070 |
| ***Abnormal reflexes*** | | | | |
| Birth weight | -0.0012 | 0.0005 | -2.25 | 0.028 |

**(B) Neonates with CBL<10μg/dL**

| NBAS Cluster | Coefficient | SE | T | P |
| --- | --- | --- | --- | --- |
| ***Motor*** | | | | |
| Head circumference | 0.48 | 0.25 | 1.93 | 0.059 |
| Maternal medical problem | -1.82 | 0.94 | -1.93 | 0.058 |
| Birth weight | 0.0029 | 0.0011 | 2.80 | 0.007 |
| Maternal obstetric problem | -2.52 | 0.86 | -2.93 | 0.005 |
| ***Range of state*** | | | | |
| Maternal obstetric problem | 1.45 | 0.80 | 1.82 | 0.074 |
| Duration of house paint | -1.27 | 0.53 | -2.38 | 0.020 |
| Birth weight | -0.0019 | 0.0009 | -2.06 | 0.044 |
| ***Autonomic stability*** | | | | |
| Hours of birth | 0.06 | 0.03 | 2.05 | 0.045 |
| Maternal medical problem | -1.82 | 1.04 | -1.75 | 0.082 |
| Maternal obstetric problem | -2.30 | 0.94 | -2.45 | 0.016 |
| ***Abnormal reflexes*** | | | | |
| Birth weight | -0.0013 | 0.0005 | -2.33 | 0.023 |
| Maturity | -0.23 | 0.12 | -1.89 | 0.064 |
| Maternal obstetric problem | 0.84 | 0.47 | 1.79 | 0.079 |

**Supplementary Table 6A.** Results of SEM analysis using the MIMIC model depicted in Figure 2A.

(A) All Neonates

|  | ***All neonates*** | | | | |
| --- | --- | --- | --- | --- | --- |
|  | **Estimate** | | **SE** | **z** | **p** |
| **Regression Coefficients** | Unstandardized | Standardized |  |  |  |
| Head circumference --> Neonatal behavior | 1.000 | 0.514 |  |  |  |
| Birth Weight --> Neonatal behavior | -0.002 | -0.174 | 0.001 | -1.258 | 0.208 |
| Blood lead --> Neonatal behavior | 0.192 | 0.586 | 0.065 | 2.958 | 0.003 |
| Maturity --> Neonatal behavior | -0.722 | -0.602 | 0.241 | -2.990 | 0.003 |
| Neonatal behavior --> Motor | 0.020 | 0.023 | 0.067 | 0.302 | 0.763 |
| Neonatal behavior --> Autonomic stability | -0.288 | -0.359 | 0.085 | -3.406 | 0.0007 |
| Neonatal behavior --> Abnormal reflexes | 0.178 | 0.368 | 0.052 | 3.450 | 0.0006 |
| Neonatal behavior --> Range of state | -0.101 | -0.109 | 0.073 | -1.387 | 0.166 |
| **Covariances** |  |  |  |  |  |
| Motor <---> Autonomic stability | 2.884 |  | 0.888 | 3.248 | 0.001 |
| Abnormal reflexes <---> Range of state | 0.022 |  | 0.532 | 0.041 | 0.967 |
| Autonomic stability <---> Range of state | 2.306 |  | 0.905 | 2.547 | 0.011 |
| Motor <---> Range of state | 2.370 |  | 1.071 | 2.214 | 0.027 |
| Autonomic stability <---> Abnormal reflexes | -0.687 |  | 0.437 | -1.572 | 0.116 |
| Motor <---> Abnormal reflexes | -2.122 |  | 0.540 | -3.928 | 0.0001 |
| **Variances** |  |  |  |  |  |
| Birth Weight | 169726.691 |  | 18144.55 | 9.354 | <1x10^-22^ |
| Head circumference | 4.507 |  | 0.482 | 9.351 | <1x10^-22^ |
| Maturity | 11.844 |  | 1.266 | 9.355 | <1x10^-22^ |
| Blood lead | 159.043 |  | 17.002 | 9.354 | <1x10^-22^ |
| Motor | 13.531 |  | 1.447 | 9.351 | <1x10^-22^ |
| Autonomic stability | 9.583 |  | 1.024 | 9.358 | <1x10^-22^ |
| Abnormal reflexes | 3.440 |  | 0.368 | 9.348 | <1x10^-22^ |
| Range of state | 14.413 |  | 1.541 | 9.353 | <1x10^-22^ |
|  |  |  |  |  |  |
| **Indices of model fit** | Test Model | Ind Model |  |  |  |
| Chi-square | 97.226 | 181.965 |  |  |  |
| Degree of freedom | 15 | 28 |  |  |  |
| Root mean squared residual | 97.123 | 97.845 |  |  |  |
| Goodness of fit index | 0.872 | 0.781 |  |  |  |
| Adjusted goodness of fit index | 0.692 | 0.719 |  |  |  |
| Parsimony goodness of fit index | 0.363 | 0.608 |  |  |  |
| Normed fit index | 0.466 | 0.000 |  |  |  |
| Relative fit index | 0.003 | 0.000 |  |  |  |
| Incremental fit index | 0.508 | 0.000 |  |  |  |
| Tucker-Lewis index | 0.003 | 0.000 |  |  |  |
| Comparative fit index | 0.466 | 0.000 |  |  |  |
| Parsimony ratio | 0.536 | 1.000 |  |  |  |
| Parsimony adjusted normed fit index | 0.249 | 0.000 |  |  |  |
| Parsimony adjusted comparative fit index | 0.250 | 0.000 |  |  |  |
| Noncentrality parameter | 82.226 | 153.965 |  |  |  |
| Minimum discrepancy (F_min_) | 0.556 | 1.040 |  |  |  |
| Root mean squared error of approximation | 0.177 | 0.177 |  |  |  |
| Akaike information criterion | 139.226 | 197.965 |  |  |  |
| Browne-Cudeck Criterion | 141.503 | 198.832 |  |  |  |
| Bayes information criterion | 205.806 | 223.329 |  |  |  |
| Consistent Akaike information criterion | 226.806 | 231.329 |  |  |  |
| Hoelter index (alpha=0.05) | 45.000 | 40.000 |  |  |  |
| Hoelter index (alpha=0.01) | 56.000 | 47.000 |  |  |  |

**Supplementary Table 6B.** Results of SEM analysis using the MIMIC model depicted in Figure 2A.

**(B) Neonates with CBL < 10 μg/dL**

|  | ***Neonates with CBL < 10 μg/dL*** | | | | |
| --- | --- | --- | --- | --- | --- |
|  | **Estimate** | | **SE** | **z** | **p** |
| **Regression Coefficients** | Unstandardized | Standardized |  |  |  |
| Head circumference --> Neonatal behavior | 1.000 | 0.694 |  |  |  |
| Birth Weight --> Neonatal behavior | 0.001 | 0.077 | 0.001 | 0.475 | 0.635 |
| Blood lead --> Neonatal behavior | -1.194 | -0.648 | 0.406 | -2.942 | 0.003 |
| Maturity --> Neonatal behavior | 0.271 | 0.305 | 0.156 | 1.736 | 0.083 |
| Neonatal behavior --> Motor | 0.397 | 0.342 | 0.109 | 3.659 | 0.0003 |
| Neonatal behavior --> Autonomic stability | 0.181 | 0.176 | 0.085 | 2.124 | 0.0340 |
| Neonatal behavior --> Abnormal reflexes | 0.021 | 0.034 | 0.048 | 0.427 | 0.6690 |
| Neonatal behavior --> Range of state | 0.356 | 0.290 | 0.110 | 3.231 | 0.001 |
| **Covariances** |  |  |  |  |  |
| Motor <---> Autonomic stability | 2.934 |  | 0.887 | 3.309 | 0.0009 |
| Abnormal reflexes <---> Range of state | -0.031 |  | 0.556 | -0.056 | 0.956 |
| Autonomic stability <---> Range of state | 1.728 |  | 0.933 | 1.853 | 0.064 |
| Motor <---> Range of state | 1.032 |  | 0.997 | 1.035 | 0.3 |
| Autonomic stability <---> Abnormal reflexes | -1.197 |  | 0.488 | -2.454 | 0.014 |
| Motor <---> Abnormal reflexes | -2.307 |  | 0.547 | -4.221 | 0.0001 |
| **Variances** |  |  |  |  |  |
| Birth Weight | 165714.965 |  | 18469.87 | 8.972 | <1x10^-22^ |
| Head circumference | 4.729 |  | 0.527 | 8.973 | <1x10^-22^ |
| Maturity | 12.477 |  | 1.391 | 8.970 | <1x10^-22^ |
| Blood lead | 2.892 |  | 0.322 | 8.981 | <1x10^-22^ |
| Motor | 11.695 |  | 1.303 | 8.975 | <1x10^-22^ |
| Autonomic stability | 10.085 |  | 1.124 | 8.972 | <1x10^-22^ |
| Abnormal reflexes | 3.658 |  | 0.408 | 8.966 | <1x10^-22^ |
| Range of state | 13.598 |  | 1.516 | 8.970 | <1x10^-22^ |
|  |  |  |  |  |  |
| **Indices of model fit** | Test Model | Ind Model |  |  |  |
| Chi-square | 85.459 | 168.353 |  |  |  |
| Degree of freedom | 15 | 28 |  |  |  |
| Root mean squared residual | 89.499 | 94.762 |  |  |  |
| Goodness of fit index | 0.894 | 0.779 |  |  |  |
| Adjusted goodness of fit index | 0.746 | 0.715 |  |  |  |
| Parsimony goodness of fit index | 0.373 | 0.606 |  |  |  |
| Normed fit index | 0.492 | 0.000 |  |  |  |
| Relative fit index | 0.052 | 0.000 |  |  |  |
| Incremental fit index | 0.541 | 0.000 |  |  |  |
| Tucker-Lewis index | 0.063 | 0.000 |  |  |  |
| Comparative fit index | 0.498 | 0.000 |  |  |  |
| Parsimony ratio | 0.536 | 1.000 |  |  |  |
| Parsimony adjusted normed fit index | 0.264 | 0.000 |  |  |  |
| Parsimony adjusted comparative fit index | 0.267 | 0.000 |  |  |  |
| Noncentrality parameter | 70.459 | 140.353 |  |  |  |
| Minimum discrepancy (F_min_) | 0.531 | 1.046 |  |  |  |
| Root mean squared error of approximation | 0.171 | 0.176 |  |  |  |
| Akaike information criterion | 127.459 | 184.353 |  |  |  |
| Browne-Cudeck Criterion | 129.946 | 185.301 |  |  |  |
| Bayes information criterion | 192.298 | 209.054 |  |  |  |
| Consistent Akaike information criterion | 213.298 | 217.054 |  |  |  |
| Hoelter index (alpha=0.05) | 48.000 | 40.000 |  |  |  |
| Hoelter index (alpha=0.01) | 58.000 | 47.000 |  |  |  |

Test model indicates the MIMIC model shown in Figure 2A while Ind Model refers to the Independence model where all the variables in the model are assumed to be independent of each other.

**Supplementary Table 7A.** Model fit indices for 11 nested models for all study subjects. Pale blue columns represent models including CBL as a predictor while columns in orange represent models excluding CBL as a covariate.

| **Model fit Index** | **Model** | **Covariates in the nested models, A=CBL, B=Maturity, C=Head circumference, D=Birth Weight** | | | | | | | | | | |
| --- | --- | --- | --- | --- | --- | --- | --- | --- | --- | --- | --- | --- |
|  |  | **A,B,C,D** | **A,B,C** | **A,C,D** | **A,B,D** | **B,C,D** | **A,B** | **A,C** | **A,D** | **B,C** | **B,D** | **C,D** |
| *CMIN/DF* | Tested | 6.482 | 6.359 | 6.213 | 3.406 | 9.891 | 3.140 | 3.849 | 4.266 | 12.536 | 4.092 | 9.984 |
|  | Independence | 6.499 | 6.714 | 6.196 | 5.181 | 7.481 | 6.017 | 5.967 | 5.477 | 7.742 | 5.631 | 7.032 |
| *RMR* | Tested | 97.123 | 1.458 | 108.597 | 85.239 | 90.571 | 1.663 | .2.214 | 99.011 | 0.815 | 76.040 | 101.282 |
|  | Independence | 97.843 | 2.951 | 106.002 | 91.798 | 109.576 | 3.252 | 3.053 | 99.029 | 1.787 | 104.087 | 120.750 |
| *GFI* | Tested | 0.872 | 0.911 | 0.920 | 0.955 | 0.884 | 0.979 | 0.975 | 0.970 | 0.924 | 0.973 | 0.939 |
|  | Independence | 0.781 | 0.804 | 0.821 | 0.831 | 0.782 | 0.836 | 0.854 | 0.858 | 0.809 | 0.838 | 0.827 |
| *AGFI* | Tested | 0.692 | 0.725 | 0.750 | 0.860 | 0.638 | 0.890 | 0.868 | 0.842 | 0.599 | 0.859 | 0.682 |
|  | Independence | 0.719 | 0.739 | 0.761 | 0.774 | 0.710 | 0.770 | 0.796 | 0.802 | 0.733 | 0.774 | 0.758 |
| *PGFI* | Tested | 0.363 | 0.293 | 0.296 | 0.307 | 0.284 | 0.186 | 0.186 | 0.185 | 0.176 | 0.185 | 0.179 |
|  | Independence | 0.608 | 0.603 | 0.616 | 0.623 | 0.587 | 0.597 | 0.610 | 0.613 | 0.578 | 0.599 | 0.591 |
| *NFI* | Tested | 0.466 | 0.594 | 0.570 | 0.718 | 0.433 | 0.861 | 0.828 | 0.792 | 0.568 | 0.806 | 0.621 |
|  | Independence | 0.000 | 0.000 | 0.000 | 0.000 | 0.000 | 0.000 | 0.000 | 0.000 | 0.000 | 0.000 | 0.000 |
| *RFI* | Tested | 0.003 | 0.053 | -0.003 | 0.343 | -0.322 | 0.478 | 0.355 | 0.221 | -0.619 | 0.273 | -0.420 |
|  | Independence | 0.000 | 0.000 | 0.000 | 0.000 | 0.000 | 0.000 | 0.000 | 0.000 | 0.000 | 0.000 | 0.000 |
| *IFI* | Tested | 0.508 | 0.635 | 0.613 | 0.783 | 0.460 | 0.901 | 0.867 | 0.833 | 0.588 | 0.846 | 0.646 |
|  | Independence | 0.000 | 0.000 | 0.000 | 0.000 | 0.000 | 0.000 | 0.000 | 0.000 | 0.000 | 0.000 | 0.000 |
| *TLI* | Tested | 0.003 | 0.062 | -0.003 | 0.425 | -0.372 | 0.573 | 0.426 | 0.271 | -0.711 | 0.332 | -0.489 |
|  | Independence | 0.000 | 0.000 | 0.000 | 0.000 | 0.000 | 0.000 | 0.000 | 0.000 | 0.000 | 0.000 | 0.000 |
| *CFI* | Tested | 0.466 | 0.598 | 0.570 | 0.753 | 0.412 | 0.886 | 0.847 | 0.806 | 0.544 | 0.822 | 0.603 |
|  | Independence | 0.000 | 0.000 | 0.000 | 0.000 | 0.000 | 0.000 | 0.000 | 0.000 | 0.000 | 0.000 | 0.000 |
| *PRATIO* | Tested | 0.536 | 0.429 | 0.429 | 0.429 | 0.429 | 0.267 | 0.267 | 0.267 | 0.267 | 0.267 | 0.267 |
|  | Independence | 1.000 | 1.000 | 1.000 | 1.000 | 1.000 | 1.000 | 1.000 | 1.000 | 1.000 | 1.000 | 1.000 |
| *NCP* | Tested | 82.226 | 48.231 | 46.921 | 21.650 | 80.023 | 8.562 | 11.398 | 13.062 | 46.143 | 12.366 | 35.936 |
|  | Independence | 153.965 | 119.986 | 109.107 | 87.807 | 136.106 | 75.260 | 74.507 | 67.162 | 101.128 | 69.463 | 90.486 |
| *FMIN* | Tested | 0.556 | 0.327 | 0.320 | 0.175 | 0.509 | 0.072 | 0.088 | 0.097 | 0.287 | 0.094 | 0.228 |
|  | Independence | 1.040 | 0.806 | 0.743 | 0.622 | 0.898 | 0.516 | 0.511 | 0.469 | 0.664 | 0.483 | 0.603 |
| *RMSEA* | Tested | 0.177 | 0.175 | 0.173 | 0.117 | 0.225 | 0.111 | 0.128 | 0.137 | 0.257 | 0.133 | 0.227 |
|  | Independence | 0.177 | 0.181 | 0.172 | 0.155 | 0.192 | 0.169 | 0.168 | 0.160 | 0.196 | 0.163 | 0.186 |
| *AIC* | Tested | 136.226 | 95.231 | 93.921 | 68.650 | 127.023 | 46.562 | 49.398 | 52.062 | 84.143 | 50.366 | 73.936 |
|  | Independence | 197.965 | 154.986 | 144.107 | 122.807 | 171.106 | 102.260 | 101.507 | 94.162 | 128.128 | 96.463 | 117.486 |
| *BCC* | Tested | 141.503 | 97.051 | 95.742 | 70.471 | 128.844 | 47.978 | 50.814 | 52.479 | 85.559 | 51.783 | 75.353 |
|  | Independence | 198.832 | 155.656 | 144.777 | 123.478 | 171.777 | 102.760 | 102.007 | 94.662 | 128.628 | 96.963 | 117.986 |
| *BIC* | Tested | 205.806 | 155.470 | 154.160 | 128.890 | 187.262 | 100.460 | 103.296 | 104.961 | 138.041 | 104.265 | 127.834 |
|  | Independence | 223.329 | 177.179 | 166.300 | 145.001 | 193.300 | 121.283 | 120.530 | 113.185 | 147.151 | 115.486 | 136.508 |
| *CAIC* | Tested | 226.806 | 174.470 | 173.160 | 147.890 | 206.262 | 117.460 | 120.296 | 121.961 | 155.041 | 121.265 | 144.834 |
|  | Independence | 231.329 | 184.179 | 173.300 | 152.001 | 200.300 | 127.283 | 126.530 | 119.185 | 153.151 | 121.486 | 142.508 |
| *ECVI* | Tested | 0.796 | 0.544 | 0.537 | 0.392 | 0.726 | 0.266 | 0.282 | 0.292 | 0.481 | 0.288 | 0.422 |
|  | Independence | 1.131 | 0.886 | 0.823 | 0.702 | 0.978 | 0.584 | 0.580 | 0.538 | 0.732 | 0.551 | 0.671 |
| *Hoelter 0.05* | Tested | 45 | 52 | 53 | 97 | 34 | 133 | 108 | 98 | 34 | 102 | 42 |
|  | Independence | 40 | 41 | 44 | 53 | 37 | 49 | 49 | 54 | 38 | 52 | 42 |

The full forms for the abbreviations are asprovided in Supplementary Table 6.

**Supplementary Table 7B.** Model fit indices for 11 nested models for neonates with CBL < 10 mg/dL. Pale blue columns represent models including CBL as a predictor while columns in orange represent models excluding CBL as a covariate.

| **Model fit Index** | **Model** | **Covariates in the nested models, A=CBL, B=Maturity, C=Head circumference, D=Birth Weight** | | | | | | | | | | |
| --- | --- | --- | --- | --- | --- | --- | --- | --- | --- | --- | --- | --- |
|  |  | **A,B,C,D** | **A,B,C** | **A,C,D** | **A,B,D** | **B,C,D** | **A,B** | **A,C** | **A,D** | **B,C** | **B,D** | **C,D** |
| *CMIN/DF* | Tested | 5.697 | 5.488 | 4.416 | 2.267 | 8.549 | 2.072 | 0.975 | 2.320 | 10.885 | 2.946 | 8.858 |
|  | Independence | 6.013 | 6.294 | 5.785 | 4.647 | 7.104 | 5.478 | 5.709 | 4.777 | 7.540 | 5.601 | 6.821 |
| *RMR* | Tested | 89.499 | 0.955 | 100.990 | 58.289 | 84.762 | 0.317 | 0.213 | 36.950 | 0.794 | 65.687 | 102.196 |
|  | Independence | 94.762 | 1.695 | 100.768 | 87.109 | 107.396 | 1.747 | 1.438 | 90.899 | 1.918 | 100.508 | 116.290 |
| *GFI* | Tested | 0.894 | 0.932 | 0.938 | 0.967 | 0.890 | 0.983 | 0.992 | 0.981 | 0.933 | 0.979 | 0.940 |
|  | Independence | 0.779 | 0.801 | 0.820 | 0.830 | 0.770 | 0.835 | 0.853 | 0.859 | 0.794 | 0.824 | 0.816 |
| *AGFI* | Tested | 0.746 | 0.787 | 0.806 | 0.896 | 0.657 | 0.912 | 0.956 | 0.901 | 0.648 | 0.888 | 0.687 |
|  | Independence | 0.715 | 0.735 | 0.761 | 0.773 | 0.693 | 0.769 | 0.794 | 0.803 | 0.712 | 0.754 | 0.742 |
| *PGFI* | Tested | 0.373 | 0.299 | 0.301 | 0.311 | 0.286 | 0.187 | 0.189 | 0.187 | 0.178 | 0.186 | 0.179 |
|  | Independence | 0.606 | 0.601 | 0.615 | 0.622 | 0.577 | 0.597 | 0.609 | 0.614 | 0.567 | 0.589 | 0.583 |
| *NFI* | Tested | 0.492 | 0.626 | 0.673 | 0.791 | 0.484 | 0.899 | 0.954 | 0.870 | 0.615 | 0.860 | 0.654 |
|  | Independence | 0.000 | 0.000 | 0.000 | 0.000 | 0.000 | 0.000 | 0.000 | 0.000 | 0.000 | 0.000 | 0.000 |
| *RFI* | Tested | 0.052 | 0.128 | 0.237 | 0.512 | -0.203 | 0.622 | 0.829 | 0.514 | -0.444 | 0.474 | -0.299 |
|  | Independence | 0.000 | 0.000 | 0.000 | 0.000 | 0.000 | 0.000 | 0.000 | 0.000 | 0.000 | 0.000 | 0.000 |
| *IFI* | Tested | 0.541 | 0.672 | 0.727 | 0.871 | 0.515 | 0.945 | 1.000 | 0.922 | 0.638 | 0.903 | 0.680 |
|  | Independence | 0.000 | 0.000 | 0.000 | 0.000 | 0.000 | 0.000 | 0.000 | 0.000 | 0.000 | 0.000 | 0.000 |
| *TLI* | Tested | 0.063 | 0.152 | 0.286 | 0.653 | -0.237 | 0.761 | 1.000 | 0.650 | -0.511 | 0.577 | -0.350 |
|  | Independence | 0.000 | 0.000 | 0.000 | 0.000 | 0.000 | 0.000 | 0.000 | 0.000 | 0.000 | 0.000 | 0.000 |
| *CFI* | Tested | 0.498 | 0.637 | 0.694 | 0.851 | 0.470 | 0.936 | 1.000 | 0.907 | 0.597 | 0.887 | 0.640 |
|  | Independence | 0.000 | 0.000 | 0.000 | 0.000 | 0.000 | 0.000 | 0.000 | 0.000 | 0.000 | 0.000 | 0.000 |
| *PRATIO* | Tested | 0.536 | 0.429 | 0.429 | 0.429 | 0.429 | 0.267 | 0.267 | 0.267 | 0.267 | 0.267 | 0.267 |
|  | Independence | 1.000 | 1.000 | 1.000 | 1.000 | 1.000 | 1.000 | 1.000 | 1.000 | 1.000 | 1.000 | 1.000 |
| *NCP* | Tested | 70.459 | 40.396 | 30.747 | 11.399 | 67.942 | 4.287 | 0.000 | 5.281 | 39.539 | 7.784 | 31.431 |
|  | Independence | 140.353 | 111.171 | 100.482 | 76.589 | 128.180 | 67.168 | 70.634 | 56.655 | 98.096 | 69.015 | 87.316 |
| *FMIN* | Tested | 0.531 | 0.307 | 0.247 | 0.127 | 0.478 | 0.051 | 0.024 | 0.058 | 0.270 | 0.073 | 0.220 |
|  | Independence | 1.046 | 0.821 | 0.755 | 0.606 | 0.927 | 0.510 | 0.532 | 0.445 | 0.702 | 0.522 | 0.636 |
| *RMSEA* | Tested | 0.171 | 0.167 | 0.146 | 0.089 | 0.217 | 0.082 | 0.000 | 0.091 | 0.248 | 0.110 | 0.221 |
|  | Independence | 0.176 | 0.181 | 0.172 | 0.151 | 0.195 | 0.167 | 0.171 | 0.153 | 0.202 | 0.169 | 0.190 |
| *AIC* | Tested | 127.459 | 87.396 | 77.747 | 58.399 | 114.942 | 42.287 | 37.899 | 43.281 | 77.539 | 45.784 | 69.431 |
|  | Independence | 184.353 | 146.171 | 135.482 | 111.589 | 163.180 | 94.168 | 97.634 | 83.655 | 125.096 | 96.015 | 114.316 |
| *BCC* | Tested | 129.946 | 89.382 | 79.734 | 60.339 | 116.929 | 43.832 | 39.444 | 44.827 | 79.084 | 47.329 | 70.976 |
|  | Independence | 185.301 | 146.903 | 136.214 | 112.321 | 163.912 | 94.714 | 98.179 | 84.200 | 125.641 | 96.500 | 114.862 |
| *BIC* | Tested | 192.298 | 146.060 | 136.411 | 117.063 | 173.606 | 94.776 | 90.388 | 95.770 | 130.028 | 98.273 | 121.920 |
|  | Independence | 209.054 | 167.784 | 157.096 | 133.202 | 184.793 | 112.694 | 116.159 | 102.181 | 143.621 | 114.541 | 132.842 |
| *CAIC* | Tested | 213.298 | 165.060 | 155.411 | 136.063 | 192.606 | 111.776 | 107.388 | 112.770 | 147.028 | 115.273 | 138.920 |
|  | Independence | 217.054 | 174.784 | 164.096 | 140.202 | 191.793 | 118.694 | 122.159 | 108.181 | 149.621 | 120.541 | 138.842 |
| *ECVI* | Tested | 0.792 | 0.543 | 0.483 | 0.363 | 0.714 | 0.263 | 0.235 | 0.269 | 0.482 | 0.284 | 0.431 |
|  | Independence | 1.145 | 0.908 | 0.842 | 0.693 | 1.014 | 0.585 | 0.606 | 0.520 | 0.777 | 0.596 | 0.710 |
| *Hoelter 0.05* | Tested | 48 | 56 | 69 | 134 | 36 | 185 | 392 | 165 | 36 | 130 | 44 |
|  | Independence | 40 | 40 | 44 | 54 | 36 | 49 | 47 | 57 | 36 | 48 | 40 |

The full forms for the abbreviations are asprovided in Supplementary Table 6.
